# Developing a Video Game as an Awareness and Research Tool Based on SARS-CoV-2 Epidemiological Dynamics and Motivational Perspectives

**DOI:** 10.1155/2023/8205408

**Published:** 2023-02-24

**Authors:** Alexis Messina, Michael Schyns, Björn-Olav Dozo, Vincent Denoël, Romain Van Hulle, Anne-Marie Etienne, Stéphanie Delroisse, Olivier Bruyère, Vincent D'Orio, Sébastien Fontaine, Michèle Guillaume, Anne-Catherine Lange, Gilles Louppe, Fabienne Michel, Anne-Sophie Nyssen, Fabrice Bureau, Eric Haubruge, Anne-Françoise Donneau, Laurent Gillet, Claude Saegerman

**Affiliations:** ^1^Department of Culture Media and Communication, Faculty of Philosophy and Letters, Video Game Research Laboratory (Liège Game Lab), University of Liège, Liège, Belgium; ^2^Management School of the University of Liège, Digital Business Laboratory (SIG Lab), University of Liège, Liège, Belgium; ^3^Department of Urban and Environmental Engineering, Faculty of Applied Sciences, University of Liège, Liège, Belgium; ^4^Department of Aerospace and Mechanical Engineering, Faculty of Applied Sciences, University of Liège, Liège, Belgium; ^5^Department of Psychology, Health Psychology, Faculty of Psychology Logopedics and Educational Sciences, University of Liège, Liège, Belgium; ^6^Department of Public Health Sciences, Faculty of Medicine, University of Liège, Liège, Belgium; ^7^Department of Social Sciences, Faculty of Social Sciences, University of Liège, Liège, Belgium; ^8^Center for Data Collection and Analysis and Strategically Useful Information “RADIUS” of the University of Liège, University of Liège, Liège, Belgium; ^9^Montefiore Institute of Electrical Engineering and Computer Science, Faculty of Applied Sciences, University of Liège, Liège, Belgium; ^10^Department of Psychology, Ergonomics and Intervention at Work, Faculty of Psychology, Logopedics and Educational Sciences, University of Liège, Liège, Belgium; ^11^Department of Functional Sciences, Faculty of Veterinary Medicine, University of Liège, Liège, Belgium; ^12^Gembloux Agro-Bio Tech, Faculty of Life Sciences and Bioengineering of the University of Liège, Liège, Belgium; ^13^Fundamental and Applied Research for Animal and Health (FARAH) Center, Department of Infectious and Parasitic Diseases, Faculty of Veterinary Medicine, University of Liège, Liège, Belgium

## Abstract

In mid-2020, the University of Liège (ULiège, Belgium) commissioned the ULiège Video Game Research Laboratory (Liège Game Lab) and the AR/VR Lab of the HEC-Management School of ULiège to create a serious game to raise awareness of preventive measures for its university community. This project has its origins in two objectives of the institutional policy of ULiège in response to the crisis caused by SARS-CoV-2 to raise awareness among community members of various preventive actions that can reduce the spread of the virus and to inform about the emergence and progression of a pandemic. After almost two years of design, the project resulted in the creation of SARS Wars, a decision-making management game for browsers and smartphones. This article presents the creative process of the game, specifically the integration of an adapted SEIR (susceptible-exposed-infectious-recovered) model, as well as the modeling of intercompartmental circulation dynamics in the game's algorithm, and the various limitations observed regarding the game's original missions and possibilities for future work. The SARS-CoV-2 video game project may be considered an innovative way to translate epidemiology into a language that can be used in the scope of citizen sciences. On the one hand, it provides an engaging tool and encourages active participation of the audience. On the other hand, it allows us to have a better understanding of the dynamic changes of a pandemic or an epidemic (crisis preparedness, monitoring, and control) and to anticipate potential consequences in the given parameters at set time (emerging risk identification), while offering insights for impact on some parameters on motivation (social science aspect).

## 1. Introduction

Infection with severe acute respiratory syndrome coronavirus 2 (SARS-CoV-2) induces coronavirus infectious disease 2019 (COVID-19) [1]. Since the first report on 31 December 2019, COVID-19 has resulted in high morbidity and mortality, with approximately 528 million confirmed cases and 6.3 million deaths worldwide [2]. By the end of May 2022, there were around 4.2 million cases in Belgium and 31.800 deaths, and 25.4 million vaccine doses were administered [3] out of a total of 11.569.034 individuals reported in January 2022 [4].

After two years of the COVID-19 pandemic, a lot of information was gained inside and outside the University of Liège (ULiège), and the idea to develop a serious game, named SARS Wars emerged, with the aim to popularize science and raise awareness about the fight against a pandemic. The game has been thought of in connection with current events, namely, the SARS-CoV-2 pandemic, and is the result of a strategic plan developed by the University of Liège through its interdisciplinary Risk Assessment Group.

Many management games exist for pandemics. However, due to the limited information available in the early stages of the COVID-19 pandemic, only a few already existing games like “Plague, Inc.” [5] were used as references for their ability to facilitate the understanding of disease transmission [6].

The main goal of the SARS Wars project was to develop a playful device based on scientific data for awareness raising, investigation, and teaching purposes. Real scientific data was used to design the game, and a complex algorithm centered on an epidemiological compartmental model adapted for the game was created by researchers from the University of Liège for this purpose.

## 2. Materials and Methods

### 2.1. Origins of the Game

The University of Liège (ULiège, Belgium) has set up a research and decision think tank in response to the global pandemic development back in early 2020. This group was tasked to set up and support various research projects aiming to study the functioning of the SARS-CoV-2, including regular screening of the spread of coronavirus through the development of a distribution system on a voluntary basis and analysis of salivary tests developed by ULiège, applied within the university community [7] and in Belgian nursing homes [8]; developing a compartmental SEIR (susceptible-exposed-infected-recovered) ULiège model based on the testing strategy [9]; and initiating a seroprevalence study of SARS-CoV-2 antibodies based on the analysis of salivary tests among the university population, front-line hospital staff at the University Hospital of Liège, immunocompromised patients, and others suffering from Alzheimer's disease, as well as their caregivers [10]. These projects were made possible by the investment of ULiège's GIGA interdisciplinary Biomedical Research Center and the service for data collection and analysis and strategically useful information (RADIUS) in providing extensive analytical data.

At the same time, a communication strategy was implemented to raise awareness of the need to follow several health guidelines in order to keep providing part of the minimal services of the institution. This strategy includes the regular publication of a status report on the pandemic within the university community. In addition, the development of a digital awareness tool easily accessible to all, such as a serious game based on real and verified scientific data, was quickly mentioned.

Many works testify to the communicative character of the video game medium, whether through the creation of a “fictional universe” not limited to the narrative or including the multiple channels surrounding the video game work such as the game interface, peripherals, paratext, or loading screens [11]. The same holds for the creation of the game itself through procedural rhetoric [12] or the expression of the structure through the “play” [13]. Furthermore, the metamodeling power [14] of the game is widely recognized; it draws on the real represented in discourses, works, and practices of all kinds to produce a model particular to its configuration and its medium. In other words, the video game seemed, within the framework of a communication project aimed at the university population, a medium with powerful possibilities, although its construction had to be thought out with rigor and coherence. The first project design was therefore proposed to the Liège Game Lab, a research laboratory specialized in the study of video games at the University of Liège, with the only obligation to use real scientific data.

The focus groups that followed the assignment of the project made it possible to identify which scientific data would be modeled in the game. First, it was decided that the game would offer the player a crisis management simulation in a population similar to that of a university and in the form of questions and answers based on real situations. Then, the algorithm of the game would be partly (or entirely) built on the compartmental epidemiological model of the SEIR-type built by ULiège. Finally, the situations that the players would face would be written in accordance with two variables: the infection rate (epidemiology) and the motivation of the population (social psychology and health psychology), which should increase or decrease according to the player's choices.

### 2.2. Previous Work

Previous work reports on video games being used in health science research, including impact studies on players' health behaviors and habits [15–17].

Regarding epidemiological sciences, several studies mobilize video games to trace the decisional trajectories of players. This attachment to serious games in the context of studies on epicrisis management shows an interest in the medium, which constitutes a relatively innovative approach to convey information and promote prevention [18–21]. Regarding SARS-Cov-2, several projects investigate the impact of serious games on changing players' perceptions of preventive measures [22]. For this purpose, a few games have been developed or studied, including the games *Plague Inc*. [6, 23, 24], *Escape COVID-19* [23], and *Point of Contact* [22].

However, very few serious games developed for research purposes use epidemiological data in their algorithms. A notable example is the study by Mohmmadnejad et al. [25], who used an agent-based model (ABM), developed by the University of Semnan in Iran, augmented with key concepts from the SEIR model to create the algorithm for their game *REACTION* (aka *serious game simulates coronavirus transmission*). Yet, no already created or studied video game directly cites a SEIR-like mathematical EpiModel in its algorithm, and many are built primarily on narrative simulations and simplified probabilistic methods. Therefore*, SARS Wars* remains innovative and original in adapting ULiège compartmental mathematical model into the game algorithm.

Regarding the application of such serious games in citizen science, which has been defined by Vohland et al. [26] as referring “to the active engagement of the general public in scientific research tasks (...) in which scientists and citizens collaborate to produce new knowledge for science and society,” several important works describe the main characteristics that a video game device should follow, although there is no clear consensus on these [27]. We should mention, in particular, the work of Baaden et al. [28] and the *ECSA 10 Principles of Citizen Science* [29]. Moreover, the collaborative work component of such serious games has been highlighted in the work of Djaouti et al. [30]. According to the “G/P/S” (gameplay/purpose/scope) model that the authors use to classify serious games given these three criteria, this specific type of game is characterized by its data exchange capacity. What differentiates these games from participatory science is the type of market for which they are designed (for a nonexpert audience vs. for exclusive scientific use). Thus, it is not surprising to find “citizen science games,” defined according to the terminology of Baaden et al. [28] as “games that allow people to produce and/or analyze scientific data” and which are built in a complex adequacy between entertainment and scientific objectives.

### 2.3. Prototyping of the Game

Two game structures were initially proposed, based on existing games: *Plague Inc. Evolved* [5], an apocalyptic simulation game in which the goal is to release a virus over several territories, and *Reigns* [31], a strategic card game requiring the user to manage a kingdom by responding to various situations in a trivial way (A or B), while maintaining good relations with four factions. The “Reigns-like” project was finally chosen, and the project was then entrusted to the Liège Game Lab for the gameplay and writing parts and to the AR/VR Laboratory of HEC-ULiège for the development part. The development of the game was therefore carried out asynchronously.

Several focus groups with researchers in epidemiology, health, computer, and game sciences were also conducted. The purpose of these groups was to monitor compliance with each integration of scientific modeling or theory into the algorithm in order to “balance” the game balance, referring here to the set of practices that make it possible to adjust the game to ensure its playability by players, each practice being specific to its author [32].

In September 2021, a prototype of the game was finalized, followed by a submission to student testers from ULiège. After compiling several positive results in terms of gaming experience and feelings of understanding about how a pandemic works, a first version of the game was published in the beginning of 2022.

### 2.4. Research Aspects

*SARS Wars* should be considered a research project in many respects. Its development is the result of a scientific method inspired by multidisciplinary research in game sciences, on the one hand. The members of the Liège Game Lab worked together to highlight the salient features of a management game and the various game elements that could be adapted or diverted to meet the scientific requirements of the project.

Additional research was conducted to determine the generic identity of the game so as to see which category of video games the project should fit into (i.e., simulations, strategy games, racing games, and first person shooters, among others). A genre is indeed constructed by gathering cultural objects whose characteristics are deemed similar [33]. Among these characteristics, two are particularly studied in game studies: the mechanical (gameplay) and thematic (narrative) dimensions of a game. Therefore, the creation of *SARS Wars* required the study of a game design corpus [12, 30, 34–36] in order to determine its generic identity, namely, a serious strategic management game for smartphones with a simulation dimension.

On the contrary, the various elements included in the game are the result of the research carried out on SARS-CoV-2 at the University of Liège across laboratories and disciplines, mainly: epi science, virology, social psychology, health psychology, and statistics. This fact must, however, be moderated, specifically because of the balance between the game's playful needs and the integrity of scientific data and systems.

## 3. Results

Following the “gameplay/purpose/scope” (G/P/S) classification model of Djaouti et al. [30], which is used to identify the key elements of serious games, *SARS Wars* can be summarized as follows.

First, the game is mainly built around a clear dimension of objectives to reach, which are directly inspired by the requirements from crisis management and epidemiological sciences. As a result, the game corresponds to the “game-based” or ludus classification of the G/P/S model.

Then, the objective of “message broadcasting” is clearly identified in the process of putting the player in a simulation as well as in the integration of scientific data. This message broadcasting is essentially focused on “educative,” “informative,” and “subjective” purposes. The questions of the game are built on a real and verified scientific model, which is embedded in the game mechanics. Therefore, they have a double informative and educational value in the contribution of elements reminding a contemporary context of the player, but with a verified documentation. Moreover, the “subjective” purpose is an integral part of the simulation, as it places the player in a complex position of personal choices that depend on several skills and knowledge, mainly from ludoliteracy [37] and health literacy [38, 39]. In addition, a “data exchange” purpose can be clearly identified, as the game allows the collection anonymously or under a pseudonym of game data from player activities.

Finally, the scope of the game corresponds to both the “healthcare” market, targeting the general public, and the scientific research and teaching markets, in this case, for specific audiences with clearly identified profiles.

### 3.1. Information Technology Development

The game was developed on the Unity 3D [40] development platform. It consisted in adapting our epidemiological model into game rules (gameplay), via the adaptation of a MATLAB [41], a scripted language for numerical calculation purposes, provided by the teams in charge of the model.

### 3.2. Basic Operations

**Figure 1:**
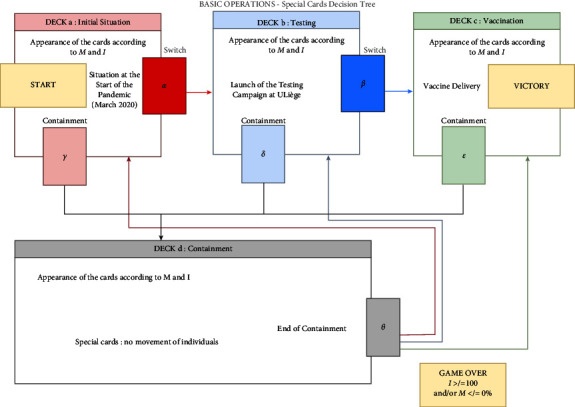
Excerpt from the game design document: basic game operations. Four thematic decks of cards are composed (a–d). Special cards (*α*, *β*, *γ*, *δ*, *ε*, and *θ*) allow the player to move from one deck to another (illustrated by directional red, blue, green, and black arrows).

*SARS Wars* is composed of four decks of cards, each containing three elements: a basic situation requiring resolution, two resolution options (answer A or B), and multipliers associated with each option (a multiplier of the *R*_0_ basic reproduction number and a multiplier of a motivational gauge). These four decks correspond to four stages of the pandemic: the initial phase (deck *a*), the testing phase (deck *b*), the vaccination phase (deck *c*), and containment (deck *d*) (see Figure 1). To move from one deck to another, the player must be able to unlock it based on several game parameters. Access to these decks is materialized by special cards: *α*, *β*, *γ*, *δ*, *ε*, and *θ*. When the players stumble upon these cards, they are offered the opportunity to move from one deck to another: *α* and *β* unlock access to decks *b* and *c*; *γ*, *δ*, and *ε* return the players to containment (deck *d*); and *θ* allows the players to move out of containment (back from *d* to *a*, *b,* or *c* depending on the previous position).

To achieve a win situation, the player must reduce the infection (gauge **I**) while maintaining a good standing with the individuals in the population, as evidenced by their willingness to follow the measures (motivation gauge **M**).

The content of the cards includes the application of proportionate or nonproportionate health measures; the organization or cancellation of events, whether discrete or mass; the communication or not of the authorities on the evolution of the pandemic; the application or not of strict measures such as lockdowns and the banning of attendance at venues; the development or not of complementary research; the arrival of new variants; dilemmas and other psychological or reasoning situations; questions of knowledge about the functioning of barrier gestures and the circulation of a virus or a vaccine; and surprises.

### 3.3. Epidemiology Model

**Figure 2:**
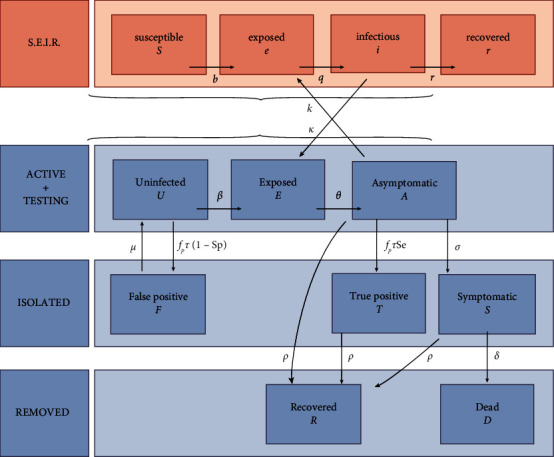
SEIR model developed by ULiège [9]. Four levels of compartments are included: the outside population, the academic population involved in the testing process, the participants who isolate themselves following a positive test, and those who leave the model (temporarily or not).

*SARS Wars* is based on a compartmental epidemiology model. This type of mathematical model implies that a targeted sample (here, the academic population) can be divided into compartments according to its condition. The elements of the sample travel from one cell to another in a specific direction. For the SARS-CoV-2 pandemic, ULiège has developed an augmented SEIR-type model [9], which considers not only the salivary testing campaign implemented by ULiège but also the circulation of individuals with the outside world (compartments colored red) (see Figure 2).

**Figure 3:**
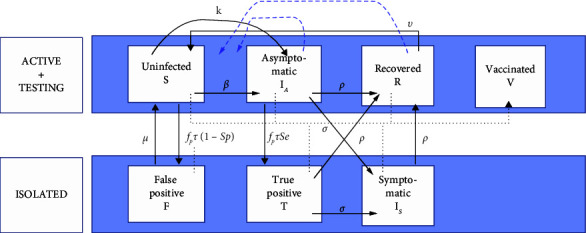
SEIR model developed by ULiège, here adapted for SARS Wars. Vaccination (thematic deck c, only one dose) makes the individuals leave the model. If vaccination is not reached, asymptomatic and recovered individuals return to the model after a period (delay of immunity following contact with the disease, illustrated here by two blue dotted arrows).

This model has been simplified to build a basic SEIR due to the large amount of data to be included in the algorithm of the game (see Figure 3). The latter excludes too much backtracking from one compartment to another when the vaccine is inoculated, thus reducing the number of doses to one. Furthermore, the involvement of a compartment composed of individuals from outside the community (home, public transport, etc.) was limited; the model operates in an almost hermetically sealed environment, where the individuals making up the population (25,000) circulate almost exclusively within the campus.

Once stripped of too much complex data, the set contains the following parameters and indicators: maximum number of infections due to the external situation at the university, automatically decreasing; time needed to cure/quarantine; loss of natural immunity; effectiveness of immunity; fraction of the population developing symptoms; return after a false positive; time to develop symptoms; sensitivity and specificity of tests and frequency of screening; participation rate in screening (from deck *b*); number of daily vaccinations (from deck *c*); and fraction of the population infected at the initial time.

A multiplier is applied to the initial *R*_0_ for each response, depending on the situation faced by the player. These multipliers range from 0.75 to 1.2 (within limits considered scientifically plausible). The higher the multiplier, the higher the *R*_0_, and the higher the infections (infection gauge = **I**). Conversely, a multiplier lower than one will reduce the *R*_0_, which eventually, depending on the evolution of the *R*_0_, may decrease the infection curve.

### 3.4. Motivational Perspective

In contrast to the epidemiological model, which predicts the evolution of the pandemic based on the actions of the player, it was impossible to create a complex motivational algorithm with the same ambitions.

During discussions with the team in charge of the behavioral model that would be complementary to the epidemiological algorithm, it was realized that both social psychology and health psychology should be mobilized jointly to simulate realistic behaviors. Indeed, the behavioral study of health can be divided into two layers. From a macroscopic point of view, elements such as socioeconomic influence, health literacy, gathering the knowledge of the individual to be able to access, understand, and evaluate information [38], or the effects of group influence on adherence to health norms [39] can be considered. From a microscopic point of view, moreover, many psychological factors can predict health behaviors, such as social, genetic, emotional, individual beliefs or health professionals' factors [42]. Thus, several theoretical models used to understand the issues of motivation for care can form the basis for constructing an algorithm, i.e., the “HBM” health beliefs model [43] or the “PMT” Protection motivation theory model [44].

However, due to the large amount of data that such models would involve, and due to the complexity of the epidemiological algorithm already created, it was decided not to integrate these models into a complex algorithm. Indeed, the more the set contains data with variable values and importance referring to scientific systems with their logic, the more difficult the balancing may be. Therefore, to keep the gameplay readable (in other words, to make it quickly recognizable and understandable in the player's aesthetic experience), it was decided to integrate elements related to the study of health behaviors only in the writing of the cards. From then on, the writing was done according to several markers: reticular interactions, socio-professional profile, social pressure, proxemics, causal attributions, belief systems, motivation, sensitivity, severity, vulnerability, threat, cost/benefit ratio, etc. For example, several situations in the game ask players to choose whether or not to organize a small or large-scale event (do you want to invite a professor? Do you want to organize a conference?) to open or close rooms on the university campus given the health situation, to organize or not press conferences to communicate on the evolution of the pandemic, or to react to the circulation of fake news. In all cases, the answers available to players remain binary: *no* or *yes*. This lack of possible reactions is unavoidable considering the complexity of the gameplay balance.

Therefore, the algorithm of the game works in a binary way. Mechanically, each response A (*no*, left finger swipe) or B (*yes*, right finger swipe) proposed to the player performs a mathematical operation to the gauge (motivation gauge = **M**), whose maximum value is 100. Depending on the initial situation, the deck itself (i.e., deck *d* relating to containment) and the nature of the responses, a subtraction or addition of values zero, five, or ten is performed. Accessorily, some situations have no impact on the gauge; these are situations where the application of health measures has no direct or perceptible effect on the motivation of the population.

### 3.5. Gameplay and Narration

The individuals (25,000) of a given population (ULiège academic community, including administrative, technical, working staff, professors and researchers, students, and rectoral authorities) circulate from one compartment to another according to their state (uninfected, asymptomatic, recovered, vaccinated, false positive, true positive, and symptomatic). This circulation depends on the player's choices, which apply a multiplier to a naturally high *R*_0_ (basic reproduction rate of a virus) at the beginning of the game, and an addition or subtraction to a gauge specific to the population's motivation.

**Figure 4:**
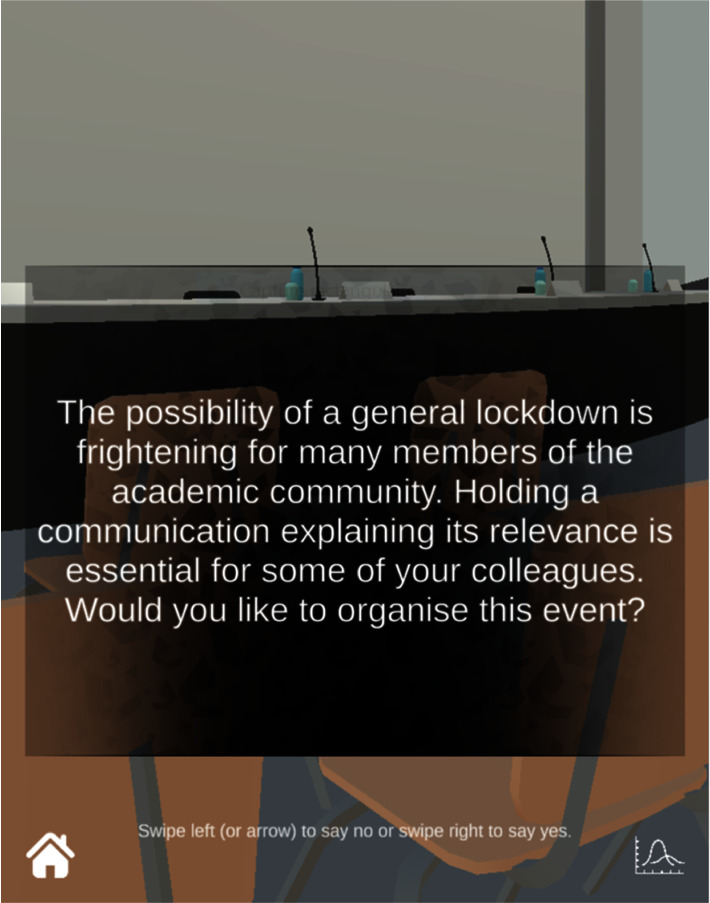
Screenshot of SARS Wars, showing a question asked during a game. The player has two choices: answer A or answer B. Each answer is associated with two values modifying both the *R*_0_ and the motivation of individuals to take health measures.

The player navigates from deck to deck (*a*, *b*, *c,* and *d*), each one representing a different stage of a pandemic, according to choices made during the game (i.e., start the testing, develop the vaccine, and enforce lockdown) (see Figure 4). In summary,

- The player makes a decision
- The player's decision applies a multiplier greater or less than 1 to the *R*_0_
- After applying the multiplier, the *R*_0_ causes the infected gauge to increase or decrease
- The player's decision also applies an addition or subtraction to the motivational gauge
- With each decision, individuals migrate from one compartment to another, with vaccination removing individuals from the model.

**Figure 5:**
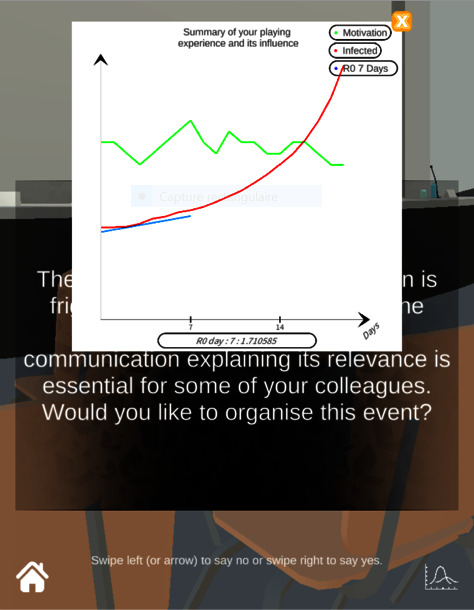
Screenshot of SARS Wars, showing the two dimensions coordinate space summarizing the status of the pandemic at each question. Three data are crossed: the evolution of the number of infected individuals (red function), the evolution of the *R*_0_ over 7 days (blue function), and the general motivation of individuals to follow the measures taken by the player (green function). These functions evolve according to the SEIR-based algorithm of the game (evolution of *R*_0_ by multiplication of a number, evolution of the motivation by addition or subtraction of a number, both defined by the choices of the players). The player is invited to read the game's vademecum (online appendix, accessible from the game's menu) to interpret more precisely the various elements of the graphic.

At any time during the game, the player can receive feedback on the evolution of the pandemic (motivation, infection, and *R*_0_ evolution over 7 days) in a two-axis reference frame (growth of the indicators over the days) (see Figure 5).

### 3.6. Data Collection

In parallel to its playable dimension, *SARS Wars* allows the collection of players' usage data on dedicated servers. The video game provides a means to compile several sets of data related to the evolution of each pandemic simulated by the player's actions: the number of situations (cards) played, the answer to each question (yes or no), the final status (victory or defeat), and the pandemic situation at each stage of its evolution (number of infected, general motivation, and *R*_0_), while the game automatically collects this data, in compliance with the European Data Protection Regulation (deactivation of data transmission in the game menu on the one hand and information about consent available on the game page and in the game on the other hand), this collection is carried out anonymously and it is not directly possible to identify the characteristics of players through the analysis of the game. The protocol aiming to collect data for a scientific study or for the collection of opinions needs to be supervised by a third party if it is to be cross-referenced with personal and identification data, or even health data, of a given population (for example, to avoid the interpretation of the data being distorted by players having completed several games, an intermediate classification of the data must be carried out by counting only the last game played). This possibility is currently being evaluated through the development of a crossover study including demographic, inclusion, infection (reported in the past), infection symptoms, vaccination symptoms, salivary testing, vaccinations, and combined vaccinations of volunteers in the SARSSURV seroprevalence study (quantitative cross-study of the simulation of a pandemic crisis management in relation to the health pathway of a cohort sensitized to the vaccine process).

## 4. Discussion

### 4.1. Limitations

The concordance between the requirements of game design and the constraints of the project for which the game is created is a recurrent theme in the research literature on scientific serious games. However, only a small number of studies highlight the specific design points that may be problematic [15, 18, 23, 25, 28]. Among these, several elements seem to be recurrent, including the adaptation of the information provided by the game. It can be observed that the complexity of the created simulation generates difficulty in the gameplay, which must be compensated by a good game design and a properly dosed information provision that must be further tailored to the target audience. This element is, moreover, identified by Baaden et al. [28] as one of the major points that must necessarily be taken into account when creating a serious game. Conversely, it also seems that each project faces specific difficulties when it comes to design issues. Therefore, a first limitation of this project is that the construction of the gameplay was done to the detriment of a strict scientific method, in order to avoid any conflict with the game's ludic functioning and its generic identity (strategy, card games, and mobile time). In this respect, Arsenault and Perron's work on the “magic cycle” of creation [45] shows that the heuristic stage in understanding the rules of the game precedes the other stages of the aesthetic experience, namely, a new heuristic stage specific to the narrative and an ultimate hermeneutic stage that concerns the entire video game object, these three steps repeating themselves endlessly in this particular order [45]. Although this conceptualization of the video game aesthetic experience must be nuanced, notably because of the prevalence of the player's playful attitude over the structure of the game in the very act of “playing” [46], importance must be given to the legibility of the gameplay and so far as it participates in the player's interpretation. In other words, a consequent simplification of the parameters of the game, including the integrity of certain scientific data, is necessary.

Moreover, the challenge of accommodating both scientific rigor and ease of use ends in failure when it comes to creating an algorithm that would consider the motivation of the model population (gauge **M**). Indeed, if the writing process respects the multiplicity of factors involved in the choice to follow or not follow sanitary measures, it also leaves a lot of room for interpretation by the writers. In addition, the association of mathematical values to the **I** (infection) and **M** (motivation) gauges for each question is done manually, according to the principle of likelihood and without precise scientific resources for each question (because these resources are not currently quantified). This association, though essentially to the gameplay, remains purely ludic and mechanical: it is the result of the subjective act of interpretation of the game authors.

It should also be added that the situations presented to the player have been deliberately written with a slant (i.e., using adjectives or adverbs qualifying a proposed epidemiological action and referring to its reception by a population, such as in the sentence “the possibility of a general lockdown is frightening for many members of the academic community,” see Figure 4). From a game mechanics point of view, the purpose of biased wording is to urge the player to action and to ground these situations in credible enunciative contexts. This naturally carries the risk of influencing the player's decisions, but it also, therefore gives us the opportunity to further investigate the effects of wording on the player by conducting additional research using more neutral terms.

Nevertheless, the results of the first beta tests show that the “subjective” purpose of the game, which consists of immersing the player in a crisis management simulation experience, seems conclusive, despite the involvement of simplified scientific data. These beta tests also show that the “informative” dimension of the game, i.e., its capacity to convey explanations on the pandemic functioning, also seems conclusive. These first beta tests were carried out informally, however, without a strict method of data collection and were focused on the question of the game's playability and its balancing. Therefore, additional reception studies are still needed to investigate the impact of simplifying the scientific elements of the game on the fulfillment of informational and subjective objectives.

### 4.2. Perspectives in Citizen Science

An essential dimension of *SARS Wars* lies in its capacity to generate models in a playful way based on nonscientific players' involvement. This process of cocreating knowledge by using video games is regularly studied in the literature on citizen science. As mentioned by Newman et al. [47], the use of new technologies is an important part of the history of open science, which was previously restricted by the geographical implementation of studies and whose participant cohorts can now reach international or continental sizes. Video games fits well into this phenomenon, as this medium is widely shared on online channels and allows for the creation of many networks.

From this perspective, *SARS Wars* meets both the criteria of Djaouti et al. [30] and the terminology of Baaden et al. [28] by allowing nonscientific players to participate in the production of knowledge; the implementation of a SEIR model in the algorithm of the game makes it possible to simulate the circulation of new viruses by modifying the base reproduction rate, the **I** and **M** values associated with each question, and even the narrative framework of the questions. Players may then simulate many possible epidemiological developments, providing data for comparative studies. Thus, a first conclusion is that the digital and plastic structure of *SARS Wars*, whose parameters can be modified, may be used by audiences outside the university.

When there is no consensus yet on the definition of the distinctive components of open science [27], some criteria serving as guidelines for examining a practice or project can be drawn from the *ECSA 10 Principles of Citizen Science* [29]. These include involving citizens in an active way to generate new knowledge (1), with a transformational or ameliorative purpose (2) from which researchers as well as citizens can benefit (3). Other points to mention are the involvement of citizens in the various stages of the scientific process at the origin of the approach (4) while giving them feedback on the project (5). Finally, a last part concerns the production of scientific data, with the consideration of the project involved as a real research project, with limitations and biases (6); the publication of data and metadata in open access (7); the recognition of citizens as coauthors of this data (8); the evaluation of the project according to scientific criteria of quality (9); and ethics, confidentiality, and environmental impact (10). According to these criteria, the postcreation review of *SARS Wars* shows a partial adequacy to the principles of citizen sciences.

As previously explained, *SARS Wars* is fully in line with the “shared production of new knowledge” criterion. The involvement of the players in this production must be nuanced nonetheless, since only the scientific supervisors of the project are able to modify the values of the game and thus propose new epidemiological parameters. Even so, this new knowledge may have a positive impact in many fields due to the plastic structure and the online sharing possibilities of *SARS Wars*. These include the management of public health policies, teaching, popularization of epidemiological dynamics, or research, provided that scientific and legal criteria are respected (points 9 and 10 of the ECSA criteria).

However, given the current postproduction stage of the *SARS Wars* project (without consideration of the reception of the game, i.e., comparative studies of the results of players from target cohorts), the above criteria cannot be adequately examined. Therefore, future work (including the SARSSURV crossstudy) will have to examine the conformity of the project's results to the following criteria: the involvement of players in the supervision of projects mobilizing the structure of *SARS Wars*, their recognition as coauthors, the publication of data and metadata concerning them in open access, and the evaluation of the transformational or ameliorative goal of the project as a whole.

## 5. Conclusions and Perspectives

Partial implementation of the SEIR ULiège model into *SARS Wars* shows that the design of video games allows for the implementation of complex models, under the condition of gameplay balancing in order not to overshadow the ludic dimension. This necessity of balancing the game is therefore an important limitation of the implementation process. Consequently, future applied research could be interested in questioning the link between respecting the necessary characteristics of a scientific model and the conditions of existence of a playful dimension. Particular attention could thus be paid to the implementation of more complex SEIR epidemiological models, but also to the construction of motivational algorithms based on the “health beliefs model” (HBM) [43] or “protection motivation theory” (PMT) [44] type models. A point of interest must be made on the impact of an oversimplification on the objective of scientific popularization, especially in the messages, and sensitization spread by the game; what needs to be questioned more deeply is essentially the aim of raising public awareness as to how a pandemic works, particularly with regard to the accuracy of the discourse carried by the game in its potential use by health professionals, scientists, and amateur audiences. Citizen involvement in the understanding and evolution of epidemiological sciences would be, through the use of serious games, an interesting avenue for future work.

In the same way, the use of serious games built on simulated models would make it possible to question several problems linked to the understanding of a pandemic as the following: the questions of crisis preparedness, monitoring, and control, the potential anticipation of emergency risks, or the impact of the parameters on the feasibility and practicality of potential decisions are all possible themes addressed by the simulated nature of the game. The main issue would be to determine to what extent the balancing of the game allows to question such themes.

Finally, a device such as *SARS Wars* seems promising as a statistical data collection device. It is indeed possible to use the most frequent trajectory used by players as a scenario for risk assessment, but also to analyze the trajectory of each player to understand the internal dynamics of the model. Thus, with a quantitative or qualitative analysis methodology, *SARS Wars* can become a platform for “directive interview” type data collection. The structure of the game can therefore be used as a teaching aid, an awareness-raising tool, or a data collection tool for research purposes.

## Data Availability

Data can be consulted on demand by contacting https://teachingwithvr.uliege.be/sarswars/web.
